# MnFe_2_O_4_-NH_2_-HKUST-1, MOF magnetic composite, as a novel sorbent for efficient dye removal: fabrication, characterization and isotherm studies

**DOI:** 10.1038/s41598-024-59727-8

**Published:** 2024-04-20

**Authors:** Masoumeh Mohammadnejad, Sedigheh Alizadeh

**Affiliations:** Department of Analytical Chemistry, Faculty of Chemistry, Alzahra University, Tehran, Iran

**Keywords:** Metal–organic framework, MnFe_2_O_4_, Magnetic adsorbent, Removal, Methylene blue, Crystal violet, Environmental chemistry, Pollution remediation, Materials chemistry, Chemical synthesis

## Abstract

Dye in industrial wastewater is one of the most serious environmental concerns due to its potentially harmful effects on human health. Many industrial dyes are carcinogenic, toxic and teratogenic. Removal and recovery of hazardous dyes from the effluents requires efficient adsorbents. In this study, magnetic adsorbent MnFe_2_O_4_-NH_2_-HKUST-1 was synthesized to remove methylene blue and crystal violet dyes from aqueous solutions. The synthesized adsorbent was characterized using FTIR, XRD, BET, VSM, SEM, TGA and Zeta potential techniques. The effect of different parameters such as pH, contact time, and adsorbent dosage on the removal of dyes was investigated. The dye adsorption process was investigated by UV–Vis spectrophotometry. The maximum adsorbent capacity was obtained as 149.25 mg/g for methylene blue and 135.13 mg/g for crystal violet. The adsorption equilibrium isotherm and kinetic models were plotted and results showed that the adsorption process for both dyes is a collection of physical and chemical adsorption based on langmuir and freundlich isotherm models, and follows the pseudo-second-order adsorption kinetics. This study shows that magnetic adsorbent MnFe_2_O_4_-NH_2_-HKUST-1 has a good potential for removal of methylene blue and crystal violet dyes from water in a short time (5 min) and it is easily separated from the solution by a magnetic field due to its magnetic property.

## Introduction

The rapid growth of the earth's population has caused the growth of the industrial revolution and its promotion. Along with the industrial revolution, more technologies and factories were developed, which have created a great negative impact on the earth's ecosystem^1^. After air, water is the most important substance needed by living organisms, on which the life and health of all organisms depend. Urban and industrial wastewaters are one of the main sources of water pollution. In the meantime, water pollution caused by textile industry effluents causes serious problems for the health of living organisms^2^.

Therefore, the effective removal of pollutants from water is very important. Various methods have been reported for water purification, including physical, biological, chemical, and electrochemical methods, as well as a combination of them^3^. The use of photocatalysts, adsorption, and membrane filtration are considered new technologies, among which adsorption has received much attention due to its cost-effectiveness, high efficiency, and simplicity^4^. For this reason, in recent years, the design and construction of new adsorbents with high adsorption and removal efficiency is increasing.

Porous metal–organic frameworks (MOFs) are one of these adsorbents that have been considered for the removal of hazardous substances due to their excellent chemical stability in the solvent, high specific surface area, adgustable pore size, and unsaturated and accessible coordination sites^5,6^. The adsorption performance of porous MOFs is directly related to their specific surface area, pore channel size, type and number of adsorption sites, electrostatic interaction with adsorbates, and π‒π interactions between benzene rings of MOF and aromatic rings of dyes and organic compounds^5,7^.

HKUST-1, which is also called Cu-BTC and MOF-199, is one of the most well-known metal–organic framework that are easy and affordable to synthesized and is very useful for different application. HKUST-1 is actually a metal–organic framework based on Copper metal^8^.

However, one of the problems related to MOF is the difficulty of collecting them from the solution. Recently, magnetic adsorbents have received special attention due to their many advantages, including easy separation, simple design, and high performance^9^. The magnetic adsorbents that used in the adsorption process can be easily collected from the wastewater by an external magnetic field. On the other hand, coupling the magnetic adsorbents with MOFs to synthesize the adsorbent increases the adsorption efficiency. In recent years, the focus of our research group has been on the synthesis of new magnetic adsorbents and efforts to optimize the efficiency and time of the adsorption process^10,11^.

Dye is one of the main sources of water pollution. Methylene blue and crystal violet are two widely used dyes in various industrie.

Methylene blue (MB) is an organic chloride salt with the molecular formula C_16_H_18_ClN_3_S, which belongs to the category of thiazine dyes. This compound was synthesized for the first time in 1876 by Heinrich Caro and it is a dark green crystalline powder and turns dark blue when dissolved in alcohol or water. This cationic dye is one of the most important water pollutants, and continuous contact with it leads to problems such as headache, confusion, vomiting, high blood pressure, shortness of breath, allergic reactions, mental disorders and other serious complications. Nevertheless, it is a widely used dye in the industry and is widely used as a coloring and disinfecting agent in medicines, pesticides, varnishes and in dyeing cotton, wood, silk, etc.^12–14^.

Crystal violet (CV) is also a cationic dye with the molecular formula C_25_H_30_ClN_3_, which is also called genetic violet. CV is a mixture of tetramethyl, pentamethyl and hexamethyl pararosanilines, and different shades of purple are obtained by combining its different types. Its solid sample is blue-green in color. This dye is widely used in the textile industry, cotton and silk dyeing, for dyeing paper and in the printing industry, as well as a pH indicator and as a skin disinfectant. CV is a water-soluble organic dye that is carcinogenic and non-degradable and stable in different environments. This dye can cause painful sensitivity to light, which in more sever cases causes permanent damage to the eye tissue. If absorbed through the skin, it causes skin irritation and digestive system irritation^15,16^.

Removal of MB and CV dye pollutants were studied with synthesized MnFe_2_O_4_-NH_2_-HKUST-1.

**Scheme 1:**
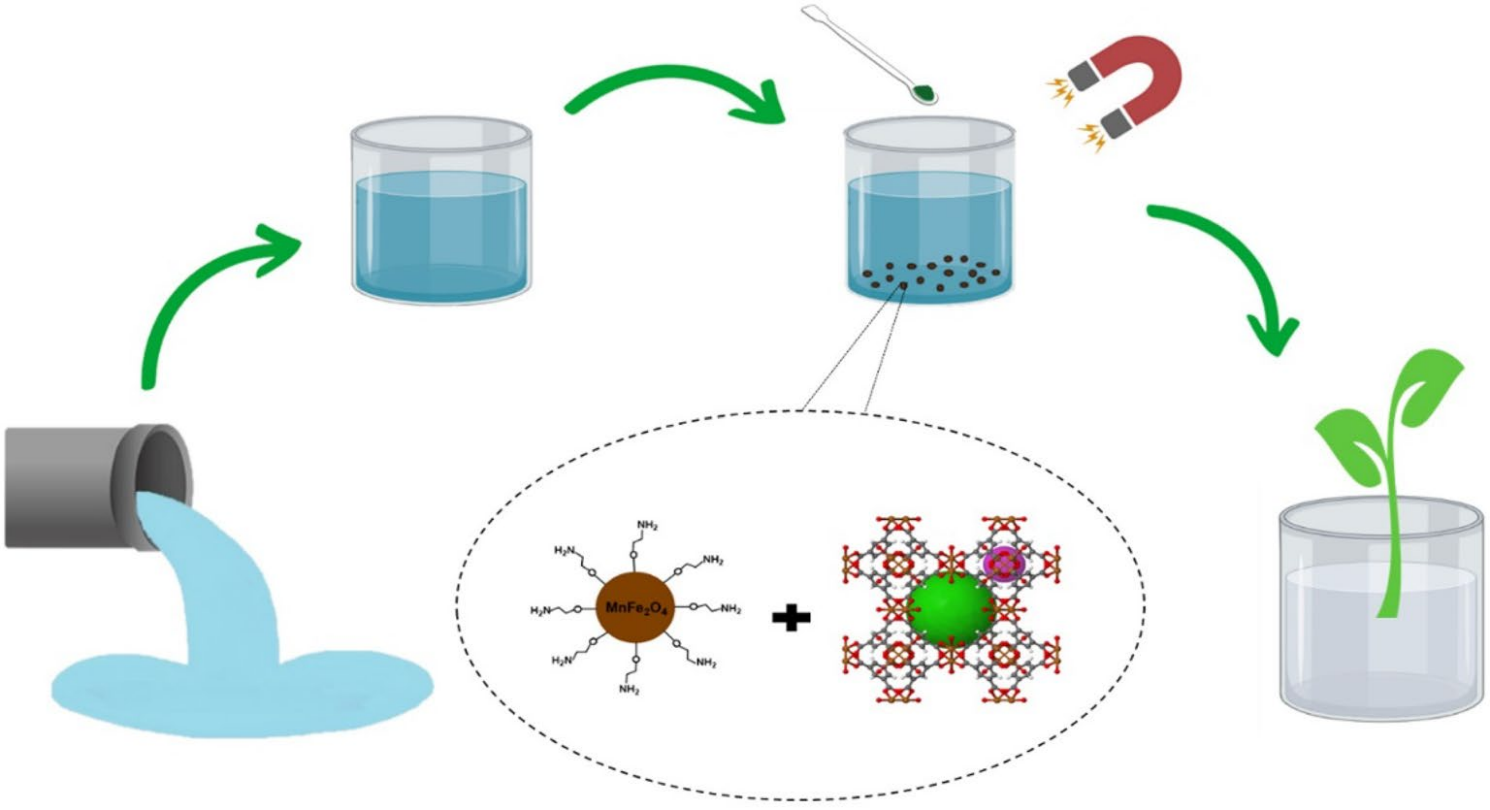
Schematic of the process.

Since MOFs possess low chemical, thermal as well as hydrothermal stability and decompose under acidic, basic or moist conditions^17^, in this research, we synthesized a novel magnetic composite with improved physical and chemical properties using a simple and efficient synthetic strategy with the combination of MnFe_2_O_4_-NH_2_ (magnetic nanoparticle) and HKUST-1 (MOF). The hybrid adsorbent developed in this study combines the advantages of metal–organic framework and magnetic nanoparticles, addressing the drawbacks associated with individual components. As a result, the synthesized adsorbent exhibits a more removal efficiency in a faster removal rate of 5 min compared to conventional adsorbents. Furthermore, its magnetic component enhances its thermal and chemical stability, enabling easy separation from the environment through an external magnetic field, aligning with modern green concepts. It is anticipated that this adsorbent can effectively remove cationic dyes, such as MB and CV, from wastewater, demonstrating its potential for pollutant purification (Scheme 1).

## Experimental

### Materials and instruments

Methylene blue (MB), Crystal violet (CV), Manganese (II) nitrate tetrahydrate Mn(NO_3_)_2_.4H_2_O, Iron (III) nitrate nonahydrate Fe(NO_3_)_3_.9H_2_O, Copper (II) nitrate trihydrate Cu(NO_3_)_2_.3H_2_O, Sodium acetate C_2_H_3_NaO_2_, Benzene—1, 3, 5-tricarboxylic acid (BTC), Ethanol C_2_H_5_OH, Ethanolamine C_2_H_7_NO, Ethylene glycol C_2_H_6_O_2_, Hydrochloric acid HCl were provided from Merck and Sigma Aldrich company.

The concentration of the dyes was determined using double-beam UV–Vis spectrophotometer (Lambda 35, Perkin-Elmer, USA). The samples were characterized by scanning electron microscopy (SEM) KYKY-EM 3200 with gold coating. pH of solutions was measured usin a glass pH electrode (metrohm 713 pH-meter). Powder X-ray diffraction (XRD) data were obtained on a Philips X'pert diffractometer with monochromatic Cu-Ka radiation.

FT-IR spectra were recorded on a Equinox 55 Bruker model FT-IR spectrophotometer using KBr pellets. Surface areas were determined by the BET (Belsorp mini II, Microtrace Bel Corop) method. The measurement of the magnetic field was done by Vibrating Sample Magnetometer (VAM LBKFB, Kavir Co). The thermogravimetric analysis (TGA) was carried out on a TGA Bahr, Germany Instrument and Zeta potential (SZ-100Z, Horiba Jobin Jyovin) analysis was done to determine the surface charge of the synthesized adsorbent.

### Synthesis of MnFe_2_O_4_-NH_2_ magnetic nanoparticle

**Scheme 2:**
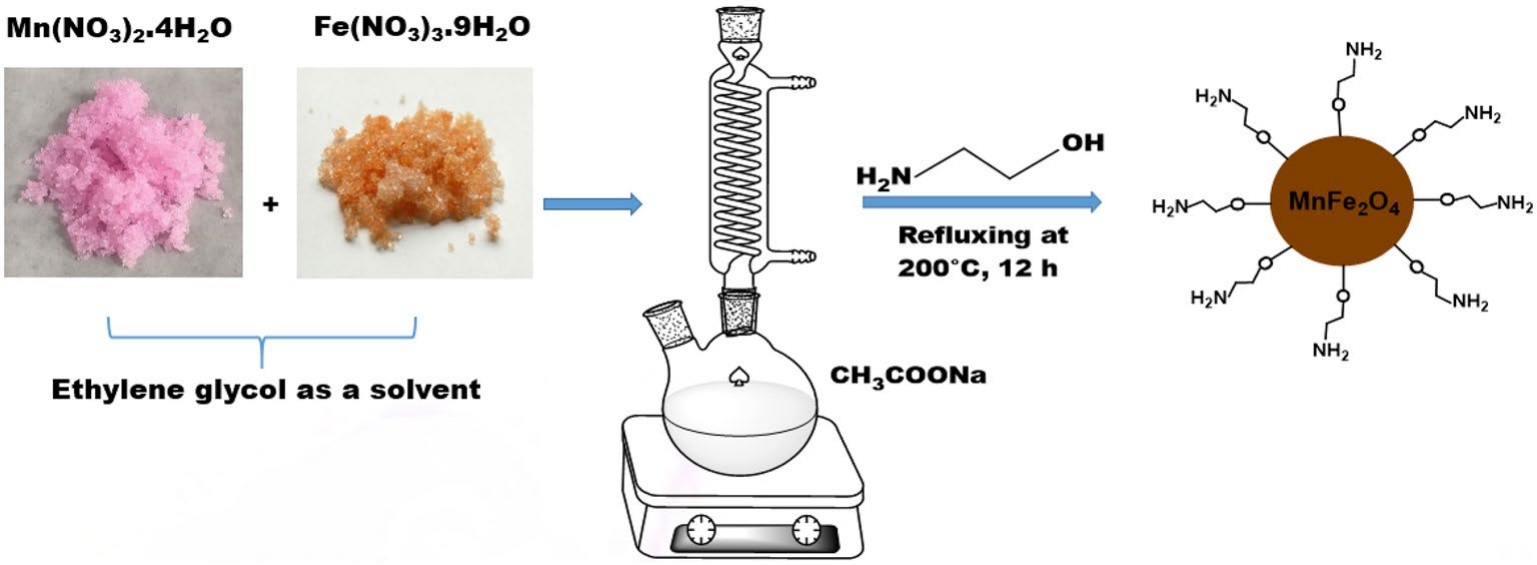
The magnetic nanoparticle synthesis process.

0.8203 g (10 mmol) of sodium acetate was dissolved in 6.5 ml of ethylene glycol, then refluxed at 100 °C for 15 min. After that, 0.1709 g (0.66 mmol) of manganese(II) nitrate tetrahydrate and 0.5385 g (1.3 mmol) of iron(III) nitrate nonahydrate were dissolved in 3.5 ml of ethylene glycol and added to the previous solution and refluxed again for 30 min. Then 2.3 ml of ethanolamine was added to the resulting mixture and refluxed for 12 h at a temperature of 200 °C. After the completion of the reaction, the obtained product, which is a brown precipitate with magnetic properties, was cooled at room temperature and separated by a magnetic separator. Then it was washed several times with water and ethanol and finally it was dried at 70 °C for 4 h (Scheme 2).

### Synthesis of MnFe_2_O_4_-NH_2_-HKUST-1 composite

**Scheme 3:**
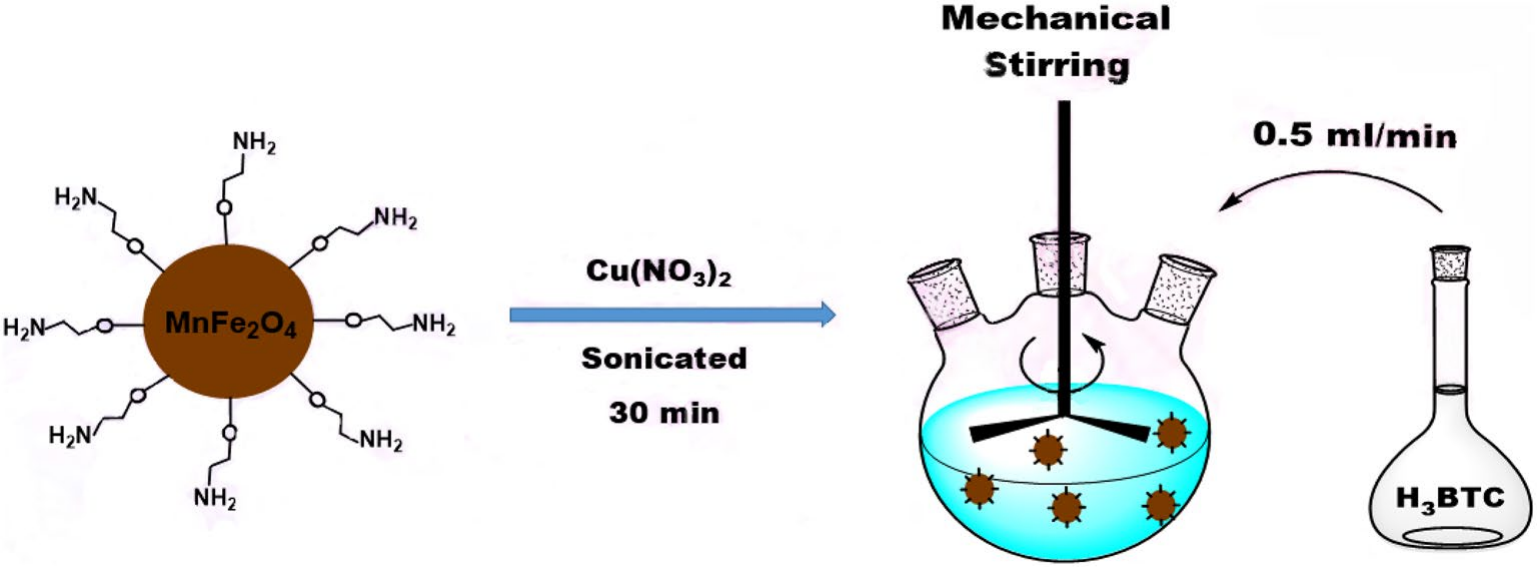
The magnetic composite synthesis process.

0.05 g of the synthesized nanoparticle (MnFe_2_O_4_-NH_2_) was dissolved with 0.4450 g (2.4 mmol) of Cu(NO_3_)_2_·3H_2_O in 25 ml of ethanol and placed in an ultrasonic bath for 30 min. After that, 0.21 g (1 mmol) of benzene 1, 3, 5 tricarboxylic acid ligand was dissolved in 25 ml of ethanol andadded to the previous solution under mechanical stirring at a rate of 0.5 ml/min for 1 h. The resulting green product was washed several times with ethanol, separated with a megnet and placed in vacuum oven at 50 °C for 4 h to dry (Scheme 3).

### Removal experiments

At first, solutions of both MB and CV dyes were prepared in the concentration range of 1.28–10.00 and 0.81–6.93 mg/l in distilled water, respectively. Then, the adsorption process at the wavelength of 664 nm for MB and 590 nm for CV was investigated by adding 5 and 3 mg of adsorbent to 5 ml solutions of these dyes. After the adsorption process was completed, the synthesized adsorbent was removed from the solutions by an external magnet and the remaining concentrations of the dyes were calculated with the following equation:

1 $$Removal\, \left(\%\right)=\left(1-\frac{A}{{A}_{0}}\right)\times 100$$

where A_0_ is the initial adsorption of the solution and A is the adsorption of the analyte after adding the sorbent.

## Result and discussion

### Characterization of MnFe_2_O_4_-NH_2_-HKUST-1 composite

Magnetic copper based MOFs namely MnFe_2_O_4_-NH_2_-HKUST-1 composites were synthesized in a simple and facile strategy, and then were characterized by SEM, EDS, FT-IR, XRD, BET, TGA, VSM and Zeta potential.

#### FTIR analysis

**Figure 1:**
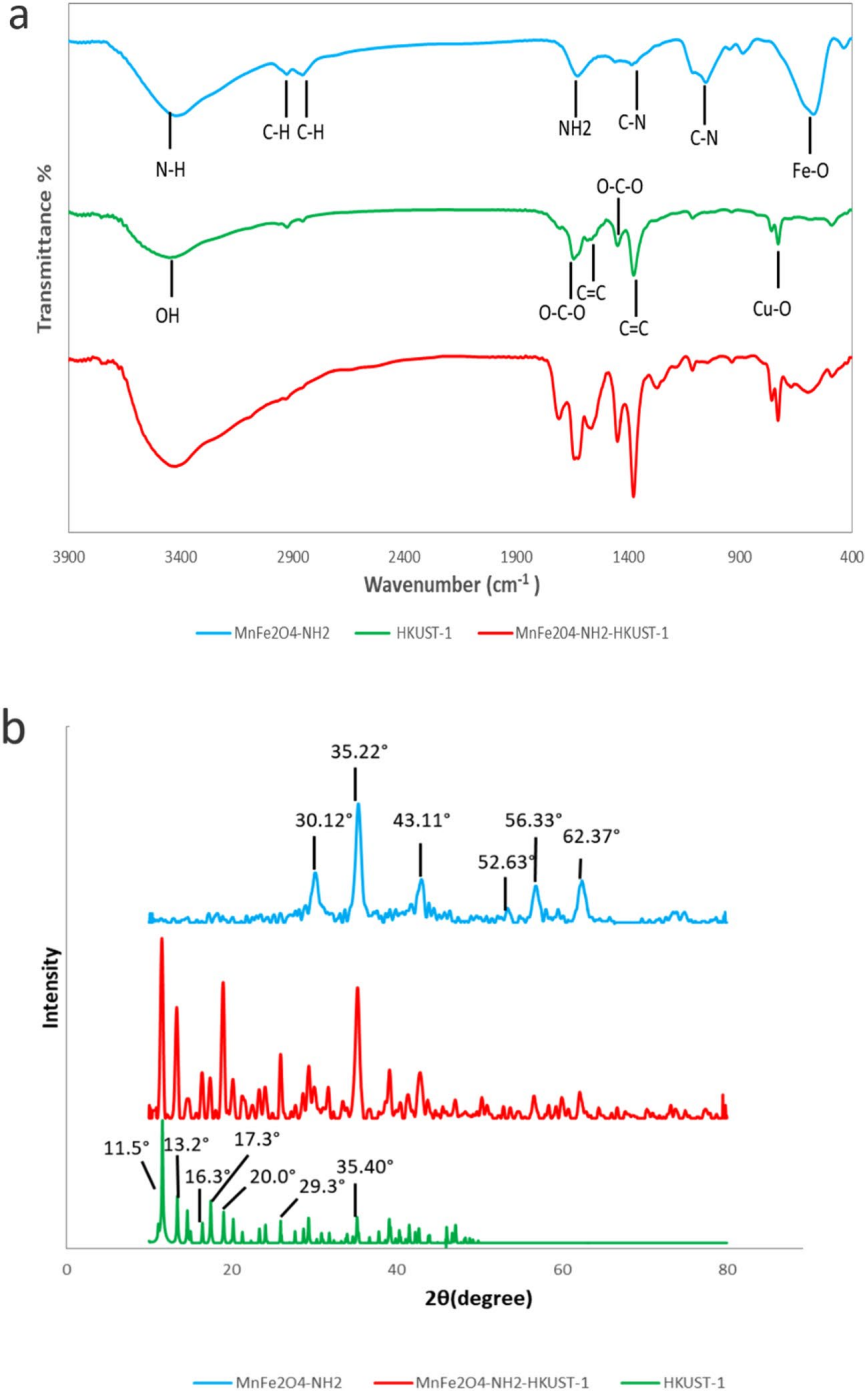
(**a**) FT-IR spectra of MnFe_2_O_4_-NH_2_, HKUST-1 and MnFe_2_O_4_-NH_2_-HKUST-1, (**b**) XRD pattern of MnFe_2_O_4_-NH_2_, MnFe_2_O_4_-NH_2_-HKUST-1 and HKUST-1.

The Fourier transform infrared (FT-IR) spectra of HKUST-1, MnFe_2_O_4_-NH_2_ and MnFe_2_O_4_-NH_2_-HKUST-1 have been shown in Fig. 1a.

In the FT-IR spectra related to HKUST-1 bands in the regions of 1447 cm^−1^ and 1639 cm^−1^ are attributed to the carboxyl groups O–C–O and the bands that appeared in the regions of 1375 cm^−1^ and 1565 cm^−1^ are attributed to the stretching vibration of C=C in the BTC ligand. The band appearing in the region of 680 cm^−1^ is related to the Cu–O bond^18^. The bands appearing in the regions of 2925 cm^−1^ and 2854 cm^−1^ are attributed to the stretching vibration of the C–H bond in ethylene glycol or ethanolamine. The broad band in the region of 1049 cm^−1^ can be attributed to the overlap of the C–O bond with the stretching vibration of the C–N bond, which indicates the binding of amino groups on the MnFe_2_O_4_-NH_2_ nanoparticle. The band in the range of 500–600 cm^−1^ is related to the Fe–O bond^19,20^. Also, the bands at 1385 cm^−1^, 1627 cm^−1^ and 3421 cm^−1^ are respectively related to C–N stretching vibration, NH_2_ scissor bending vibration and N–H stretching vibration, which is a sign of the presence of ethanolamine molecules on the nanoparticle surface^21^. The broad peak appearing in the region of 3418 cm^−1^ can be attributed to –OH water molecules in the structure of HKUST-1^11^.

The peaks in the investigated spectra (HKUST-1 and MnFe_2_O_4_-NH_2_) correspond to the infrared spectrum of the magnetic adsorbent MnFe_2_O_4_-NH_2_-HKUST-1.

#### XRD analysis

MnFe_2_O_4_-NH_2_ nanoparticles and MnFe_2_O_4_-NH_2_-HKUST-1 composite obtained from the synthesis were then characterized using XRD. In the diffractogram pattern of MnFe_2_O_4_-NH_2_ (Fig. 1b), the peaks appearing at the angles of 2θ = 30.120°, 35.225°, 43.114°, 52.63°, 56.33°, 62.372°, agree with the X-ray diffraction pattern of the reference^20^. In the diffractogram pattern of HKUST-1, the peaks in the angles of 2θ = 11.5°, 13.2°, 16.3°, 17.3°, 20°, 29.3°, 35° are the characteristic peaks of this structure^18,22^.

The peaks in the examined patterns can be found in the diffractogram of the synthesis of the MnFe_2_O_4_-NH_2_-HKUST-1 adsorbent.

#### SEM micrographs

**Figure 2:**
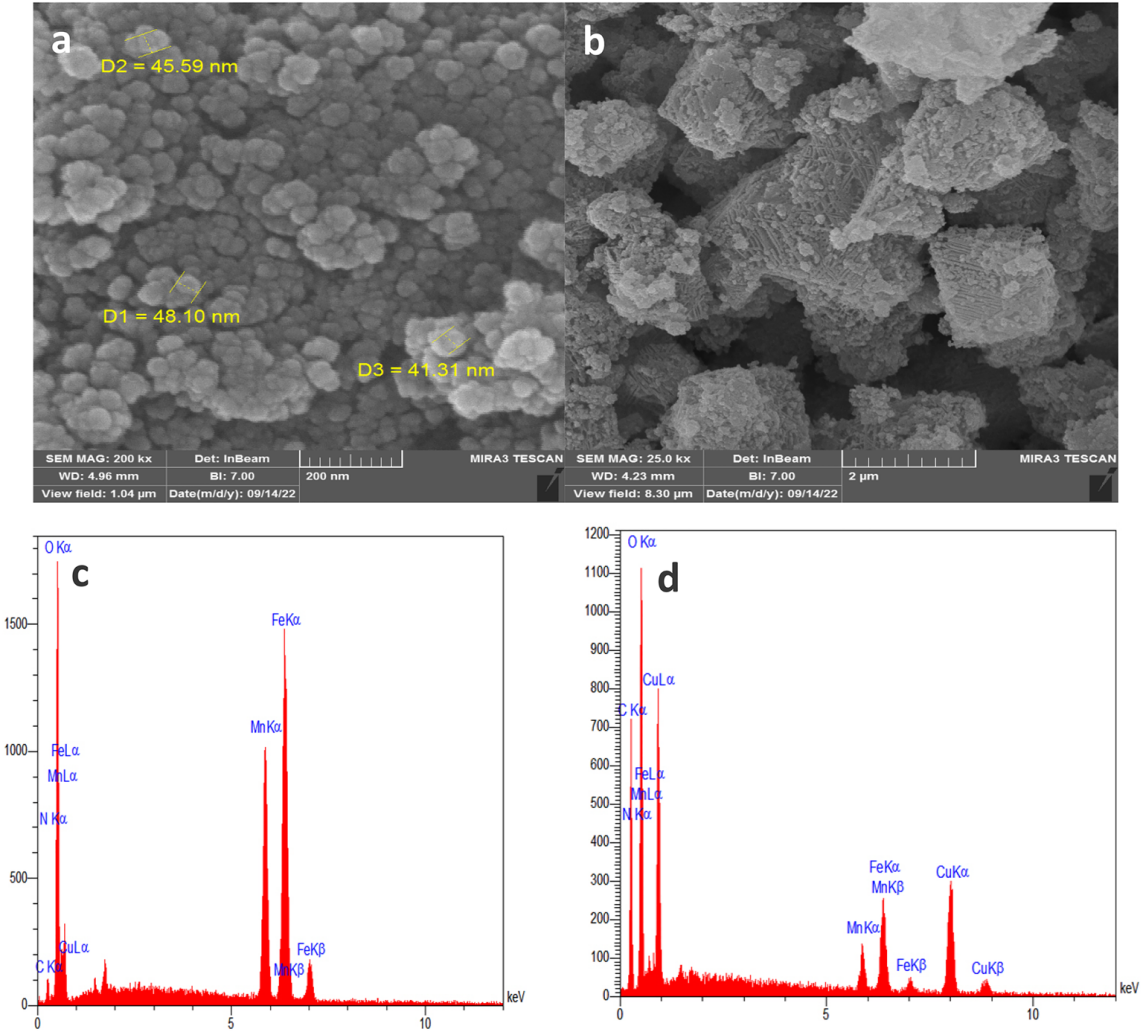
SEM micrographs of (**a**) MnFe_2_O_4_-NH_2_ magnetic nanoparticle, (**b**) MnFe_2_O_4_-NH_2_-HKUST-1 magnetic composite, EDX spectra of (**c**) MnFe_2_O_4_-NH_2_ magnetic nanoparticle and (**d**) MnFe_2_O_4_-NH_2_-HKUST-1 magnetic composite.

SEM analysis was used to determine the morphology and particle size of MnFe_2_O_4_-NH_2_ nanoparticles and magnetic adsorbent MnFe_2_O_4_-NH_2_-HKUST-1. Fig. 2a shows metal oxide nanoparticles MnFe_2_O_4_-NH_2_ in two magnifications. According to these images, metal oxide nanoparticles are spherical shape with an approximate diameter of 45 nm. Figure 2b is the recorded SEM images of MnFe_2_O_4_-NH_2_-HKUST-1 magnetic adsorbent octahedral particles, which seems that the nanoparticles are placed on the surface of HKUST-1.

#### EDX analysis

EDX analysis was used to semi-quantitatively identify the constituent elements of the sample. Figure 2c and d are the EDX analysis of MnFe_2_O_4_-NH_2_ and MnFe_2_O_4_-NH_2_-HKUST-1, respectively. The presence of oxygen, iron and manganese elements in the spectrum of MnFe_2_O_4_-NH_2_ and elements of carbon and copper in the spectrum of MnFe_2_O_4_-NH_2_-HKUST-1 in addition to the above elements are fully evident.

#### BET analysis

**Figure 3:**
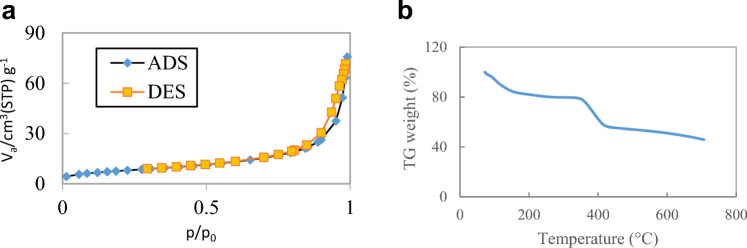
(**a**) N_2_ adsorption–desorption isotherm of MnFe_2_O_4_-NH_2_-HKUST-1 magnetic composite at 77 K, (**b**) Thermogravimetric (TG) analyses of the MnFe_2_O_4_-NH_2_-HKUST-1.

In order to determine the specific surface area of the magnetic adsorbent, the adsorption–desorption of nitrogen gas at the temperature of 77 K was used. The adsorption–desorption of N_2_ is attributed to a type IV isotherm wich shows that the adsorbent has a mesoporous structure. According to the results obtained from this analysis, the synthesized MnFe_2_O_4_-NH_2_-HKUST-1 has a specific surface area equal to 333.46 m^2^g^-1^ and average pore diameter equal to 17.234 nm (Fig. 3a).

#### TGA analysis

To determine the thermal stability of MnFe_2_O_4_-NH_2_-HKUST-1 magnetic adsorbent, TGA analysis was performed. Figure 3b shows the changes in the mass. The first weight loss is observed (20%) in the temperature range of 80-150 °C, which can be attributed to the evaporation of water and other volatile substances in the adsorbent crystal. The second weight loss in the temperature range of 150–220 °C is due to the removal of solvent molecules and other unreacted organic chemicals in the adsorbent structure, and a significant weight loss at 350°C indicates the destruction of adsorbent organic ligands. As the temperature increases, only metal oxides such as CuO and Fe_2_O_3_ remain and finally, the sample is completely decomposed.

#### VSM analysis

**Figure 4:**
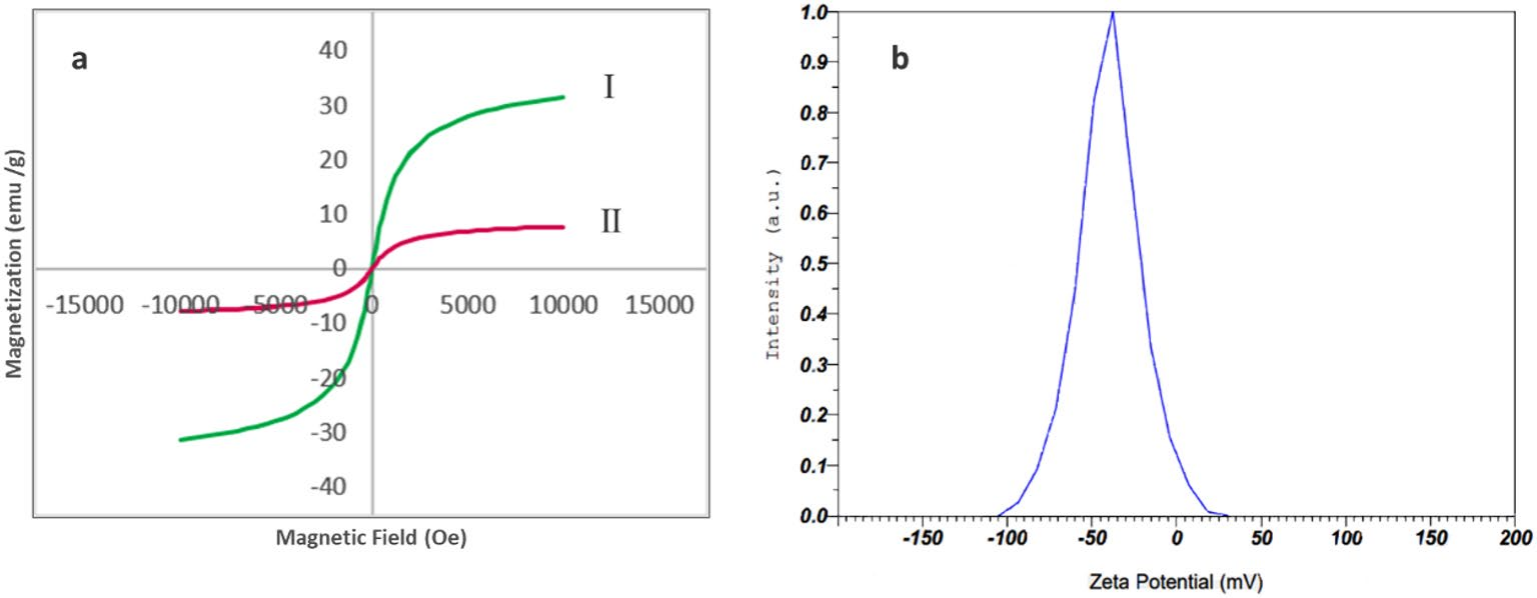
(**a**) Magnetization curves of I) MnFe_2_O_4_-NH_2_-HKUST-1, II) MnFe_2_O_4_-NH_2_-HKUST-1 and (**b**) Zeta potential of magnetic composite MnFe_2_O_4_-NH_2_-HKUST-1.

In order to determine the magnetic properties of MnFe_2_O_4_-NH_2_ and MnFe_2_O_4_-NH_2_-HKUST-1, VSM analysis was performed. Figure 4a shows the hysteresis curves of these compound. According to the graph in Fig. 4a (I), the value of saturation magnetization of the MnFe_2_O_4_-NH_2_ nanoparticle was 31.47 emu/g. Fig. 4a (II) is related to the saturation magnetization of MnFe_2_O_4_-NH_2_-HKUST-1 adsorbent, which is calculated as 7.70 emu/g and has decreased compared to the saturation magnetization of nanoparticles, which is due to the thickening of the non-magnetic component, however, the magnetic property of the synthesized adsorbent is favorable for its quick separation by magnet from the solution. According to the obtained results, the synthesized adsorbent has superparamagnetic properties and is well collected from the solution with an external magnet.

#### Zeta potential

Zeta potential analysis was used to determine the surface charge of the magnetic adsorbent. The result of the analysis is given in Fig. 4b, which shows that the surface charge of the adsorbent is negative and equal to − 39.8 mv, which according to this feature seems to have a good ability to remove positively charged species.

### Application of composite for removal of dyes

**Figure 5:**
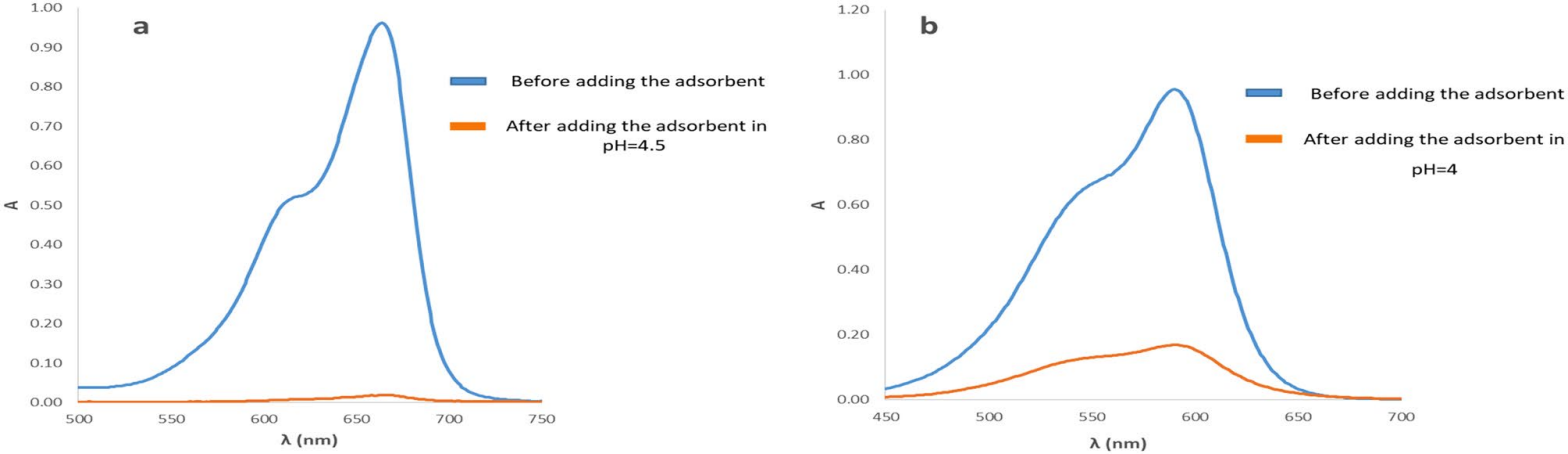
The adsorption spectrum of (**a**) MB and (**b**) CV before and after adding the adsorbent.

The efficiency of the synthesized adsorbent was checked by performing the adsorption process of two dyes, MB and CV, for this purpose, the adsorption spectrum was taken from the dyes before and after adding the adsorbent (Fig. 5a,b).

In order to increase the efficiency of the adsorption process, all parameters affecting the surface adsorption process such as solution pH, adsorbent weight and contact time were optimized.

#### Initial pH optimization

**Figure 6:**
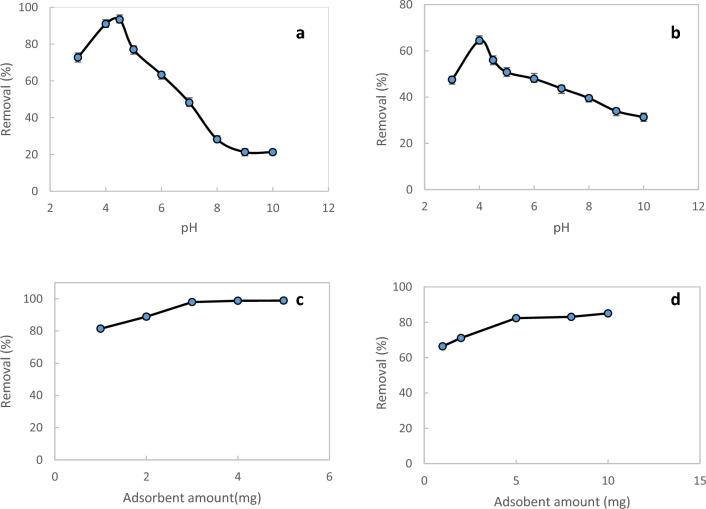
The effect of pH on the adsorption process of (**a**) MB, (**b**) CV by the sorbent in the range of 3.0 to 10 and the graph of the adsorption percentage of (**c**) MB and (**d**) CV from the solution according to the weight of the sorbent.

pH of the solution has an important effect on the interaction between the adsorbates and the adsorbents. In order to investigate the effect of this parameter, the dye adsorption process was carried out within the solution pH ranging from 3.0 to 10.0 and removal percentage was calculated based on Eq. (1) (Fig. 6a,b). The results showed that the maximum adsorption capacity of MnFe_2_O_4_-NH_2_-HKUST-1 adsorbent for MB and CV is in pH solution of 4.5 and 4, respectively. Variation in adsorption levels at different pH values can be attributed to the electrostatic interaction between the charged surface of the adsorbent and organic dyes^23^. In these pH values, the dyes are in their cationic forms and can adsorbed on the negative surface of sorbent (based on zeta potential analysis) due to the electrostatic interaction. A decrease in adsorption at high acidic media can be due to excessive protonation of the adsorbent surface, which gives it a positive charge. This leads to increased electrostatic repulsive interaction between the adsorbent surface and the positively charged MB and CV, resulting in reduced adsorption. At higher pH values, the concentration of OH^-^ ions in the solution increseas. This leads to competition between the adsorbent surface and OH^-^ ions for the MB and CV cations which results in a decrease in the adsorption.

#### Amount of sorbent optimization

A favorable adsorbent should have a suitable acceptance capacity for the analyte. In order to investigate the effect of adsorbent weight, the surface adsorption process for two dyes, MB and CV, was performed by adding 1–5 mg and 1–10 mg of adsorbent to 5 ml of 1.5 × 10^−5^ and 1.3 × 10^−5^ mol/L solution of MB and CV, respectively. The results showed that the optimal weight of the adsorbent for MB is 3 and for CV is 5 mg, which is shown in Fig. 6c and d.

#### Effect of adsorption time

Stirring time is one of the parameters affecting the adsorption process, which determines the kinetics test time. In order to determine the optimal time, the dye adsorption process was carried out at different times and resulting adsorption spectra of both dyes were examined. For both dyes, it was observed that after 5 minutes, the amount of adsorption was fixed. Therefore, this time was chosen as the optimal time for both dyes.

#### Reusability and reproducibility

Reusability studies showed that the synthesized adsorbent doesn't have much ability for repeated use and it was the major disadvantage of it. In order to check the reproducibility, four experiments were performed in optimal conditions for both MB and CV dyes. The results are reported in Table 1.

**Table 1:** Reproducibility tests of MB and CV adsorption on MnFe_2_O_4_-NH_2_-HKUST-1 composite.

Test no.	Analyte	pH	Amount of sorbent (mg)	Adsorption time (min)	% Removal	% RSD
1	MB	4.5	3	5	97.97	0.35
2					98.18	
3					97.75	
4					97.39	
1	CV	4	5	5	82.30	0.47
2					82.07	
3					82.49	
4					81.60	

#### Adsorption capacity and isotherm

Surface adsorption isotherms are used to investigate the interaction between the adsorbates and the adsorbents. In this research, four isotherms of Langmuir, Freundlich, Temkin and Dubinin–Radushkevich were studied.

##### Langmuir isotherm model

**Figure 7:**
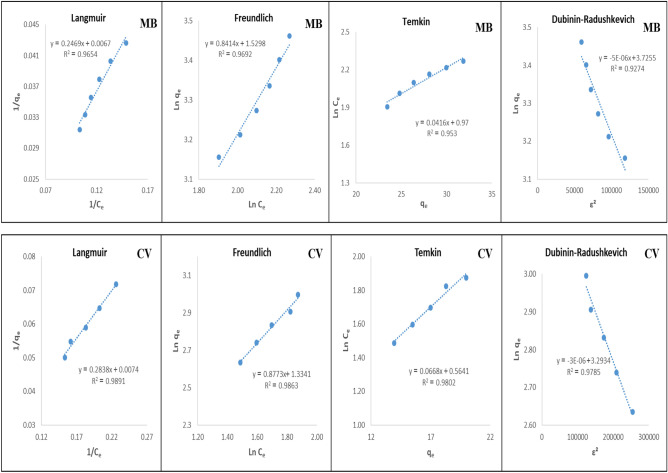
Isotherm diagrams for MB and CV.

The Langmuir isotherm was presented in 1916 by Irving Langmuir to describe surface adsorption, specially chemical surface adsorption. This model describes surface adsorption in a mono layer^24^. The linear form of the Langmuir model is given in Eq. (2):

2 $$\mathrm{Langmuir\, model}: \frac{1}{{q}_{e}}= \frac{1}{{q}_{max}{K}_{L}{C}_{e}}+\frac{1}{{q}_{max}}$$

where q_e_ (mg g^−1^) is the equilibrium adsorption capacity; C_e_ (mg L^−1^) Final concentration of MB and CV; q_max_ (mg g^−1^) is the maximum adsorption capacity; K_L_ (L mg^−1^ or L mol^−1^) Langmuir or equilibrium constant for adsorption. K_L_ and q_max_ value can be calculated from the slope and intercept of the linear plot of 1/Q_e_ versus 1/C_e_ as shown in Fig. 7.

The shape of the Langmuir model was calculated by using the separation factor R_L_, which is presented in the form of Eq. (3):

3 $${R}_{L}=\frac{1}{{1+bc}_{0}}$$

If (R_L_ = 1) isotherm is linear, (R_L_ = 0) irreversible, (R_L_ ˃ 1) unfavorable and 0 ˂ R_L_ ˂ 1 favorable

##### Freundlich isotherm model

The freundlich model is presented to describe multilayer and physical adsorption and it is assumed that the surface of the adsorber is heterogeneous and at first the adsorption sites that give a stronger bond are filled^25^. The linear mode of this model is given in Eq. (4):

4 $$Freundlich\, model: Ln {q}_{e}=Ln {K}_{f}+ \frac{1}{n} Ln {C}_{e}$$

where n and K_F_ are the Freundlich constants and express the intensity and capacity of adsorption; 1/n is the heterogeneity. Freundlich equilibrium constants were obtained from the linear plot of log Q_e_ versus log C_e_.

##### Temkin isotherm model

This model assumed that the heat of adsorption in different layers decreases linearly for all molecules due to the interactions of the adsorbate and adsorbent. The linear form of this model is given in Eq. (5):

5 $$Temkin\, model: {q}_{e}={B}_{T} Ln {A}_{T}+{B}_{T} Ln {C}_{e}$$

where B is constant related to the heat of adsorption and it is defined by the expression B = RT/b; b (J mol^−1^) is the temkin constant and indicates the adsorption temperature; T (K) absolute temperature; R (8.314 J/mol K) is the gas constant; A (L g^−1^) Temkin isotherm constant. The values B_T_ and A_T_ can be calculated from the slope and intercept of the plot of Q_e_ versus log C_e_^26^.

##### Dubinin–Radushkevich (D–R) isotherm model

In this model, it is assumed that the characteristics of the adsorption curves are related to the porosity of the adsorbent and the adsorption process occurs on heterogeneous surfaces. By using this isotherm, the type of adsorption (physical-chemical) can be recognized^27^. The linear form of this model is in the form of Eq. (6):

6 $$Dubinin-Radushkevich\, model: Ln {q}_{e}= Ln {q}_{D}-\beta {\varepsilon }^{2}$$

where q_D_ (mg g^-1^) is the maximum adsorption capacity; β is the activity coefficient useful in obtaining the mean sorption energy E (kJ mol^−1^) and ε (J mol^−1^) is the Polanyi potential which can be correlated by the following equations:

7 $$\varepsilon = RT Log \left(1+\frac{1}{{C}_{e}}\right)$$

8 $$E= \frac{1}{\sqrt{2\beta }}$$

If the value of E is less than 40 (kJ mol^−1^), the process of adsorption is physical and values higher than that indicate chemical adsorption^28^. β and Q_D_ were obtained from the slope and intercept of the plot of log Q_e_ versus ε^2^.

As shown in Fig. 7 and Table 2, it can be seen that the value of the coefficient correlation (R^2^) obtained for the Langmuir and freundlich is higher compared to other models. It could be deduced that these models are applicable in the studied concentration range. Based on the q_max_ value obtained from the Langmuir isotherm, the maximum adsorption capacity of the adsorbent for MB and CV dyes were 149.25 mg g^−1^ and 135.13 mg g^−1^, respectively. The values of R_L_ obtained from this isotherm indicate the adsorption process is favorable for both dyes. The n values derived from the freundlich equation were 1.1885 for MB and 1.1399 for CV. The n value was greater than unity which indicates the favorable adsorption. The positive values obtained for the b parameter from the temkin isotherm indicates that the adsorption occurs during an exothermic process. The calculated mean sorption energy E from the D-R isotherm for MB and CV were higher than 40 kJ mol^−1^, indicating chemical adsorption.

**Table 2:** Isotherm constants for MB and CV.

Constants	MB	CV
Langmuir equation		
R^2^	0.9654	0.9891
q_max_ (mg g^−1^)	149.25	135.13
K_L_ (L mg^−1^)	0.0271	0.0261
R_L_	0.8848	0.8785
Freundlich equation		
R^2^	0.9692	0.9863
K_F_ (mg g^−1^) (L mg^−1^) 1/n	4.6172	3.7966
n	1.1885	1.1399
Temkin equation		
R^2^	0.953	0.9802
B_T_ (J mol^−1^)	0.0416	0.0668
b_T_ (J mol^−1^)	5.96 × 10^4^	3.71 × 10^4^
Dubinin–Radushkevich equation		
R^2^	0.9274	0.9785
β (mol^2^ kJ^2−1^)	5 × 10^–6^	3 × 10^–6^
E (kJ mol^−1^)	316.2278	408.2483

These results suggest that the adsorption of MB and CV on MnFe_2_O_4_-NH_2_-HKUST-1 is a collection of physical and chemical adsorption.

#### Adsorption kinetics

Studying the kinetics of the adsorption process is necessary for a better understanding of the adsorption mechanis. In this study, two conventional kinetic models of pseudo-first-order and pseudo-second-order adsorption were investigated. Kinetic parameters were calculated from the following equations:

9 $$Log \left({q}_{e}-{q}_{t}\right)=Log {q}_{e}- \frac{{k}_{1}t}{2.303}$$

10 $$\frac{t}{{q}_{t}}=\frac{1}{{k}_{2}{q}_{e}^{2}}+ \frac{t}{{q}_{e}}$$

where q_e_ (mg g^−1^) and q_t_ (mg g^−1^) are the amount at adsorption equilibrium and the amounts of MB and CV adsorbed at time t (min), respectively, and k_1_ (min^−1^) and k_2_ (g mg^−1^ min^−1^) are the pseudo-first-order and pseudo-second-order rate constants, respectively^29^.

The values of the correlation coefficients of the models (Table 3) show that the adsorption of MB and CV on MnFe_2_O_4_-NH_2_-HKUST-1 follows the pseudo-second-order model.

**Table 3:** Pseudo-first-order and pseudo-second-order kinetic model.

Kinetic model	MB	CV
Pseudo-first-order		
k_1_ (min^−1^)	0.0334	0.4463
R^2^	0.9773	0.9748
Pseudo-second-order		
k_2_ (g mg^−1^ min^−1^)	4.7405	3.0667
R^2^	0.991	0.9994

#### Adsorption thermodynamics

In order to investigate the effect of temperature on the adsorption process of MB and CV by magnetic adsorbent and determine the thermodynamic parameters, the surface adsorption process was carried out under optimal conditions and at five different temperatures. The results showed that the removal percentage of these two dyes decreases with increasing temperature. The thermodynamic parameters of entropy change (ΔS°), free energy change (ΔG°) and enthalpy change (ΔH°) were computed with the following equations:

11 $$\Delta {G}^{0}=-RT Ln \frac{{q}_{e}}{{C}_{e}}$$

12 $$\Delta {G}^{0}=\Delta {H}^{0}-T\Delta {S}^{0}$$

where R (8.314 J mol^−1^ K) is the universal gas constant, K (L mol^−1^) is the adsorption equilibrium constant, T (K) temperature, q_e_ the amount of MB or CV adsorbed and C_e_ the MB or CV concentration in solution^30^.

The thermodynamic parameters were determined − 31975 and − 36210 (kJ mol^−1^) for ΔH and 88.395 and 107.35 (J mol^−1^ K^−1^) for ΔS for MB and CV, respectively. The calues of ΔH indicate that the adsorption process is exothermic which shows that the adsorption process is more favorable at lower temperatures.

## Conclusion

In this research, MnFe_2_O_4_-NH_2_-HKUST-1 magnetic adsorbent was synthesized and used to remove MB and CV dyes from aqueous solution. FTIR, XRD, BET, VSM, SEM, TGA and Zeta potential analyzes were performed to determine the morphology and structure of the synthesized adsorbent. Surface adsorption experiments were conducted to remove these two dyes. The effect of different factors on the adsorption process such as pH, contact time and amount of adsorbent was investigated. pH 4.5 and pH 4 were chosen as the optimal pH to remove MB and CV, respectively. The results of the experiments showed that the recovery of dye removal for each color is high in very low time, 5 min. By examining the adsorption isotherms, it was concluded that the adsorption process of this compound on the adsorbent is a collection of physical and chemical adsorption. The maximum adsorption capacity for MB and CV was 149.25 mg g^−1^ and 135.13 mg g^−1^, respectively. Thermodynamic investigations showed that the adsorption process of these dyes on the synthesized adsorbent is spontaneous and exothermic. The results of the experiments showed that the synthesized adsorbent has good magnetic properties that could be collected from the solution and has a good capacity to remove MB and CV from aqueous solutions in short time.

## Data Availability

All data generated or analyzed during this study are included in this published article.
